# Development and validation of a multi-parametric energy density optimization algorithm for microwave ablation of benign thyroid nodules: a retrospective cohort study

**DOI:** 10.3389/fendo.2026.1746874

**Published:** 2026-02-13

**Authors:** Mingfeng Mao, Ling Jiang, Xuejing Zhang, Hao Sun, Ling Lin

**Affiliations:** 1 Department of Medical Ultrasonics, The Central Hospital of Wuhan, Tongji Medical College, Huazhong University of Science and Technology, Wuhan, Hubei, China; 2 Department of Otolaryngology, The Central Hospital of Wuhan, Tongji Medical College, Huazhong University of Science and Technology, Wuhan, Hubei, China

**Keywords:** energy density, microwaves ablation techniques, optimization algorithm, precision medicine, thyroid nodule

## Abstract

**Objective:**

This study aimed to develop and validate a personalized energy density optimization algorithm for microwave ablation of benign thyroid nodules.

**Methods:**

This retrospective cohort study analyzed 82 patients undergoing MWA for benign thyroid nodules. Patients were divided into treatment success group (VRR >90%, n=31) and treatment insufficient group (VRR ≤90%, n=51) based on 12-month outcomes. *LOESS curve fitting* analysis was applied to explore the relationship between energy density and VRR at 12 months. Linear regression was used to predict optimal energy density, and logistic regression was used to estimate treatment success probability. Performance was evaluated using receiver operating characteristic (ROC) analysis (AUC), calibration assessment, and decision curve analysis. A three-step personalized energy density algorithm was established based on the regression analyses.

**Results:**

At post-ablation 12-months, 37.8%(n=31) achieving treatment success. *LOESS* curve fitting revealed a plateau effect above 4.0 J/mm^3^. The energy density prediction model incorporated vertical diameter, baseline volume, TSH, neutrophil count, and peak intensity (*adjusted R^2^ = 0.47*). Prediction model demonstrated excellent discrimination (*AUC=0.902*) with optimal cutoff probability at 0.417. Independent predictors included maximum diameter, baseline volume, WBC count, CRP, and enhancement pattern. Decision curve showed the benefit threshold was 0.8. The three-step algorithm was developed, which including baseline energy calculation, success probability estimation, and adaptive adjustment when predicted success <80%.

**Conclusions:**

Personalized energy density calculation based on patient-specific factors has the potential to significantly improve MWA outcomes for benign thyroid nodules. This algorithmic approach enables precision treatment planning and optimal patient selection.

## 1 Introduction

Thyroid nodules represent a highly prevalent clinical challenge, with detection rates reaching 65-70% in the general population when evaluated by high-frequency ultrasound (1, 2). Although more than 90% of these nodules are benign, a subset requires intervention due to compressive symptoms, cosmetic concerns, or patient anxiety regarding potential malignant transformation (2). The increasing detection of symptomatic benign thyroid nodules has driven demand for effective treatment strategies that balance therapeutic efficacy with preservation of thyroid function and patient quality of life.

Traditional surgical thyroidectomy, while definitive, carries significant morbidity including a 2.10% rebleeding rate, 0.97% nerve paresis, and 4.40% hypocalcemia at 6 months post-operatively, along with inevitable scarring and the potential need for lifelong hormone replacement therapy (3, 4). These limitations have catalyzed the development of minimally invasive ablation techniques, progressing from percutaneous ethanol injection to thermal ablation methods including laser ablation, radiofrequency ablation (RFA), and most recently, microwave ablation (MWA) (5). MWA offers distinct technical advantages including reduced treatment time, larger ablation zones, and decreased heat sink effect, making it particularly suitable for highly vascular thyroid tissue (3, 5).

Despite these theoretical advantages, clinical outcomes remain heterogeneous with concerning variability in treatment success. Recent multicenter studies report volume reduction ratios (VRR) ranging from 73% to 81% at 12–24 months, while complete nodule disappearance rates vary dramatically from 15.2% to 97.6% (4, 6). This substantial variability suggests that current “one-size-fits-all” energy delivery protocols fail to account for critical patient-specific factors. The mean energy requirement per volume reduction ranges from 0.4 to 4.6 kJ/ml, with solid nodules requiring 2.30 ± 1.5 kJ/ml compared to only 0.75 ± 0.25 kJ/ml for cystic nodules, highlighting the need for individualized energy planning (7).

The biophysical principles underlying successful ablation depend on achieving temperatures of 60-100 °C throughout the target volume while avoiding carbonization, which occurs above 105 °C and is associated with increased complications and treatment insufficient (8). Multivariate analyses have identified nodule composition, enhancement patterns, and baseline volume as independent factors affecting ablation efficacy, yet these insights have not been systematically integrated into clinical protocols (5). Furthermore, nodule location relative to critical structures, vascularity assessed by contrast-enhanced ultrasound, and patient-specific metabolic factors all influence heat distribution and ablation completeness (9).

Therefore, this study aims identifying key predictors of treatment success in microwave ablation for benign thyroid nodules, developing and validate a multivariable model for optimal energy density calculation incorporating morphological, functional, and patient-specific parameters; and establishing a clinically applicable algorithm for personalized energy delivery.

## 2 Methods

### 2.1 Study design and patient selection

This retrospective cohort study analyzed medical records of patients with benign thyroid nodules treated with microwave ablation at our institution between January 2023 and December 2023. The study protocol was approved by the institutional review board with waiver of informed consent due to its retrospective nature.

Patients with benign thyroid nodules were enrolled. Inclusion criteria were: (1) single or multiple benign thyroid nodules with maximum diameter ≥10 mm, (2) symptomatic nodules or cosmetic concerns, and (3)complete follow-up data at 3, 6, and 12 months post-ablation. Exclusion criteria included (1) suspicious or malignant cytology (Bethesda categories III-VI), (2) hyperfunctioning nodules, (3) severe coagulopathy, (4) pregnancy or lactation, and (5) incomplete follow-up data.

### 2.2 Microwave ablation procedure

All procedures were performed by experienced interventional radiologists with more than 5 years of thyroid ablation experience. Patients were positioned supine with neck hyperextension. Under ultrasound guidance, the trans-isthmic approach and hydrodissection technique were employed to protect critical structures. The moving-shot technique was utilized, with power settings ranging from 25-35W adjusted according to nodule characteristics and location. Total ablation time and energy delivered were recorded for each procedure. Energy density (J/mm^3^) was calculated as total energy delivered/baseline nodule volume.

### 2.3 Treatment efficacy evaluation and grouping

The efficacy evaluation was based on grayscale ultrasound. Post-ablation ultrasound statistics at 3, 6, and 12 months were collected and Volume reduction ratio (VRR) were calculated.

Treatment success was defined as VRR >90% at 12 months. Patients were categorized into two groups based on treatment outcome: success group (VRR >90%) and failure group (VRR ≤90%) for subsequent analysis of predictive factors.

### 2.4 Data collections

Grayscale ultrasonography characteristics (dimensions, composition, echogenicity, calcification, and location relative to the dangerous triangle) were collected at each follow-up period. Contrast-enhanced ultrasound (CEUS), laboratory assessments comprised thyroid function tests including free T3 (FT3), free T4 (FT4), thyroid-stimulating hormone (TSH), thyroglobulin (TG), anti-thyroglobulin antibody (TGAb), and anti-thyroid peroxidase antibody (TPOAb), as well as complete blood count and C-reactive protein (CRP) measurements before the ablation were collected.

### 2.5 Statistical analysis

Statistical analyses were performed using SPSS version 26.0 (IBM Corp., Armonk, NY, USA) and R version 4.3.0 (Vienna, Austria). A two-sided *P value <0.0*5 was considered statistically significant.

#### 2.5.1 Descriptive statistics and univariate analysis

Continuous variables were expressed as mean ± standard deviation for normally distributed data or median (interquartile range) for skewed distributions. Categorical variables were presented as frequencies and percentages. The *Shapiro-Wilk test* was used to assess normality. Differences between success and failure groups were analyzed using *independent t-tests* or *Mann-Whitney U tests* for continuous variables, and chi-square or Fisher’s exact tests for categorical variables. Variables with *P <0.20* in univariate analysis were considered potential predictors for inclusion in multivariable models. *Repeated measures ANOVA* was employed to evaluate temporal changes in VRR across follow-up periods.

#### 2.5.2 LOESS curve

*Locally weighted scatterplot smoothing (LOESS) regression* was applied to explore the non-linear relationship between energy density and VRR at 12 months. The optimal bandwidth was selected using *cross-validation* to identify the plateau effect threshold for energy density.

#### 2.5.3 Multivariable logistic regression analysis

To develop an optimal energy density prediction model, multivariable linear regression was performed using energy density as the dependent variable. Variables with *P <0.20* in univariate analysis were entered into the model. Stepwise backward elimination was applied with a retention criterion of *P <0.05*. Model assumptions including linearity, homoscedasticity, and normality of residuals were verified. Variance inflation factors (*VIF*) were calculated to assess multicollinearity.

#### 2.5.4 Multivariable logistic regression analysis

Multivariable logistic regression model was constructed to predict treatment success (VRR >90% at 12 months). Candidate predictors identified from univariate analysis were entered using forward stepwise selection. Model performance was evaluated using the area under the receiver operating characteristic (ROC) curve (*AUC*). The optimal cutoff probability was determined using the Youden index. Sensitivity, specificity, positive predictive value (PPV), and negative predictive value (NPV) were calculated at the optimal threshold.

#### 2.5.5 Model validation and algorithm development

Internal validation was performed using bootstrap resampling (*1000 iterations*) to assess model stability and optimism-corrected performance metrics. Calibration was evaluated using the *Hosmer-Lemeshow goodness-of-fit test* and *calibration plot* comparing predicted versus observed probabilities. Decision curve analysis was conducted to evaluate the clinical utility across different threshold probabilities.

Based on the validated models, a three-step personalized algorithm was developed: (1) baseline energy density calculation using the linear regression equation, (2) success probability estimation using the logistic regression model, and (3) adaptive energy adjustment based on predicted success probability. The algorithm was implemented as an interactive web-based calculator for clinical application. Correlation heatmaps were generated to visualize relationships between variables and treatment outcomes.

## 3 Results

### 3.1 Baseline characteristics and treatment parameters

A total of 82 patients with benign thyroid nodules underwent microwave ablation. The median baseline nodule volume was 2655.0 mm^3^ (IQR: 123.5-11165.6). Most nodules were solid (70.7%). Patient demographics, nodule characteristics, and baseline thyroid function tests are summarized in **Table 1**. The volume reduction ratio demonstrated significant temporal evolution across follow-up periods (*P < 0.001*, *repeated measures ANOVA*, **Supplementary Table 1**). Mean VRR reaching 83.6 ± 12.0% at 12 months, and treatment success (defined as *VRR >90% at 12 months*) was achieved in 31 patients (37.8%), with none achieving this threshold at 3 or 6 months (**Supplementary Table 1**).

**Table 1:** Baseline characteristics of patients undergoing microwave ablation for thyroid nodules (n=82).

Characteristics	Value
Demographics	
Age (years)^a^	49.2 ± 12.3
Female sex, n (%)	72 (87.8)
BMI (kg/m²)^a^	22.3 ± 4.2
Nodule characteristics	
Volume (mm³)^b^	2655.0 (123.5-11165.6)
Maximum diameter (mm)^a^	19.0 ± 13.8
Solitary nodule, n (%)	31 (37.8)
Calcification present, n (%)	24 (29.3)
Location in dangerous triangle, n (%)	35 (42.7)
Thyroid function tests	
TSH (mIU/L)^b^	1.30 (1.01-1.86)
FT3 (pmol/L)^a^	4.49 ± 0.62
FT4 (pmol/L)^a^	13.31 ± 1.79
Comorbidities	
Hypertension, n (%)	14 (17.1)
Coronary artery disease, n (%)	2 (2.4)
Diabetes mellitus, n (%)	0 (0.0)

^a^Data are presented as mean ± standard deviation.

^b^Data are presented as median (interquartile range).

BMI, body mass index; TSH, thyroid-stimulating hormone; FT3, free triiodothyronine; FT4, free thyroxine.

**Figure 1:**
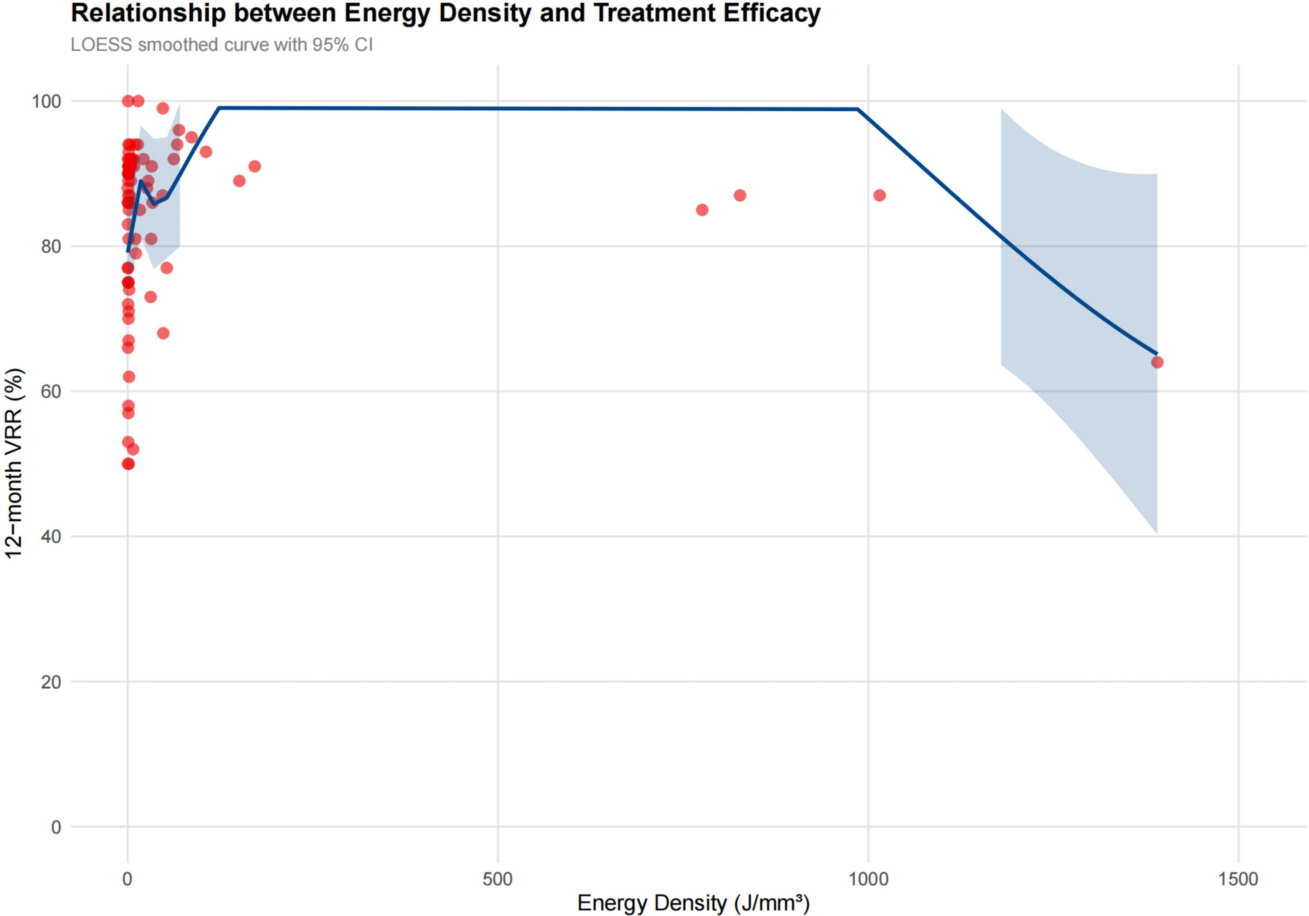
LOESS curve fitting between energy density and treatment efficacy.

The median energy density delivered was 2.19 J/mm^3^ (IQR: 0.99-24.89). Analysis of energy density distribution revealed a non-linear relationship with treatment outcomes. Scatter plot analysis with *LOESS curve* fitting suggested a plateau effect above approximately 4.0 J/mm^3^, indicating that excessive energy delivery may not provide additional benefit (**Figure 1**).These findings suggested the one-size-fits-all energy density approach was insufficient.

### 3.2 Predictors of treatment success and prediction models

#### 3.2.1 Univariate analysis for predictor identification

To identify potential predictors for our models, we performed univariate analysis of over 40 baseline variables (**Supplementary Table 2**). Nine variables met our initial screening criterion (*P < 0.20*) for model inclusion (**Table 2**) including nodule dimensional parameters, laboratory Parameters, imaging features and CEUS enhancement Patterns.

**Table 2:** Significant univariate predictors of treatment success (P < 0.20).

Variable	Total (n=82)	Insufficient Group (n=51)	Success Group (n=31)	P-value
Nodule Characteristics				
Maximum diameter (mm)	19.02 ± 13.81	21.82 ± 14.99	14.41 ± 10.24	**0.017**
Transverse diameter (mm)	15.81 ± 12.22	18.49 ± 13.42	11.41 ± 8.42	**0.010**
Longitudinal diameter (mm)	16.62 ± 12.74	19.39 ± 14.17	12.06 ± 8.34	**0.011**
Vertical diameter (mm)	13.39 ± 9.78	14.93 ± 10.18	10.85 ± 8.66	0.066
Baseline volume (mm³)	8804.91 ± 14253.30	11985.10 ± 16685.37	3572.99 ± 6248.34	**0.009**
Laboratory Parameters				
Lymphocyte count (×10^9^/L)	9.99 ± 15.25	7.89 ± 13.12	13.43 ± 17.92	0.111
CRP (mg/L)	4.05 ± 4.32	3.51 ± 3.35	4.93 ± 5.51	0.151
Imaging Features				
Calcification present, n (%)	24 (29.3)	18 (35.3)	6 (19.4)	0.198
CEUS Enhancement Pattern, n (%)				**0.004**
- Hypoenhancement	20 (24.4)	7 (13.7)	13 (41.9)	
- No enhancement	7 (8.5)	7 (13.7)	0 (0.0)	
- Isoenhancement	26 (31.7)	20 (39.2)	6 (19.4)	
- Hyperenhancement	29 (35.4)	17 (33.3)	12 (38.7)	

Data are presented as mean ± standard deviation or n (%) unless otherwise specified.

Bold values indicate statistical significance (P < 0.05).

Success defined as VRR >90% at 12 months.

Complete univariate analysis of all 40+ variables available in **Supplementary Table S1**.

CRP, C-reactive protein; CEUS, contrast-enhanced ultrasound; VRR, volume reduction ratio.

#### 3.2.2 Energy density prediction model

Building on univariate findings, we developed a multivariable linear regression model to predict optimal energy density. Through stepwise selection from candidate predictors, five independent variables were retained in the final model: vertical diameter (*β = -6.44, P < 0.001*), baseline volume (*β = 0.007, P = 0.001*), TSH level (*β = 13.75, P = 0.044*), neutrophil count (*β = 0.44, P = 0.009*), and peak intensity (representing peak contrast enhancement on CEUS) (*β = -3.22, P = 0.037*). This model explained 52% of the variance in optimal energy density (*adjusted R² = 0.47, F = 13.89, P < 0.001*), providing the foundation for individualized energy calculation (**Table 3**).

**Table 3:** Multivariable linear regression model for optimal energy density prediction.

Variable	β Coefficient	SE	t-value	P-value
Intercept	121.27	37.46	3.24	0.003
Vertical diameter (mm)*	-6.44	1.34	-4.81	<0.001
Baseline volume (mm³)	0.007	0.002	3.71	0.001
TSH (mIU/L)	13.75	6.49	2.12	0.044
Neutrophil count (×10^9^/L)	0.44	0.16	2.83	0.009
Peak intensity	-3.22	1.46	-2.20	0.037

Model R², 0.52; Adjusted R², 0.47; F-statistic, 13.89; P < 0.001.

SE, standard error; TSH, thyroid-stimulating hormone.

*Vertical diameter, the nodule diameter perpendicular to the MWA probe axis.

#### 3.2.3 Treatment success prediction model

Parallel to energy optimization, we developed a multivariable logistic regression model to predict treatment success probability. The final model incorporated both morphological and laboratory parameters: maximum diameter (*OR = 1.94, 95% CI: 1.15-3.29, P = 0.013*), baseline volume (*OR = 1.00, 95% CI: 0.99-1.00, P = 0.014)*, WBC count (*OR = 0.54, 95% CI: 0.31-0.93, P = 0.028*), and CRP level (*OR = 1.38, 95% CI: 1.09-1.73, P = 0.007*). Enhancement pattern remained highly predictive, with isoenhancement showing markedly reduced odds of success compared to hypoenhancement (*OR = 0.06, 95% CI: 0.01-0.41, P = 0.004*) (**Table 4**).

**Table 4:** Multivariable logistic regression model for treatment success prediction.

Variable	β Coefficient	OR (95% CI)	P-value
Intercept	1.83	6.26 (0.26-148.99)	0.257
Maximum diameter (mm)	0.66	1.94 (1.15-3.29)	0.013
Transverse diameter (mm)	-0.26	0.77 (0.58-1.02)	0.074
Longitudinal diameter (mm)	-0.33	0.72 (0.51-1.01)	0.059
Baseline volume (mm³)	-0.0004	1.00 (0.99-1.00)	0.014
WBC count (×10^9^/L)	-0.62	0.54 (0.31-0.93)	0.028
CRP (mg/L)	0.32	1.38 (1.09-1.73)	0.007
Enhancement pattern*			
- No enhancement	-18.46	<0.001	0.993
- Isoenhancement	-2.83	0.06 (0.01-0.41)	0.004
- Hyperenhancement	-0.82	0.44 (0.07-2.89)	0.393

*Reference category, Hypoenhancement.

Bold values indicate statistical significance (P < 0.05).

OR, odds ratio; CI, confidence interval; WBC, white blood cell; CRP, C-reactive protein.

#### 3.2.4 Model validation and performance assessment

**Figure 2:**
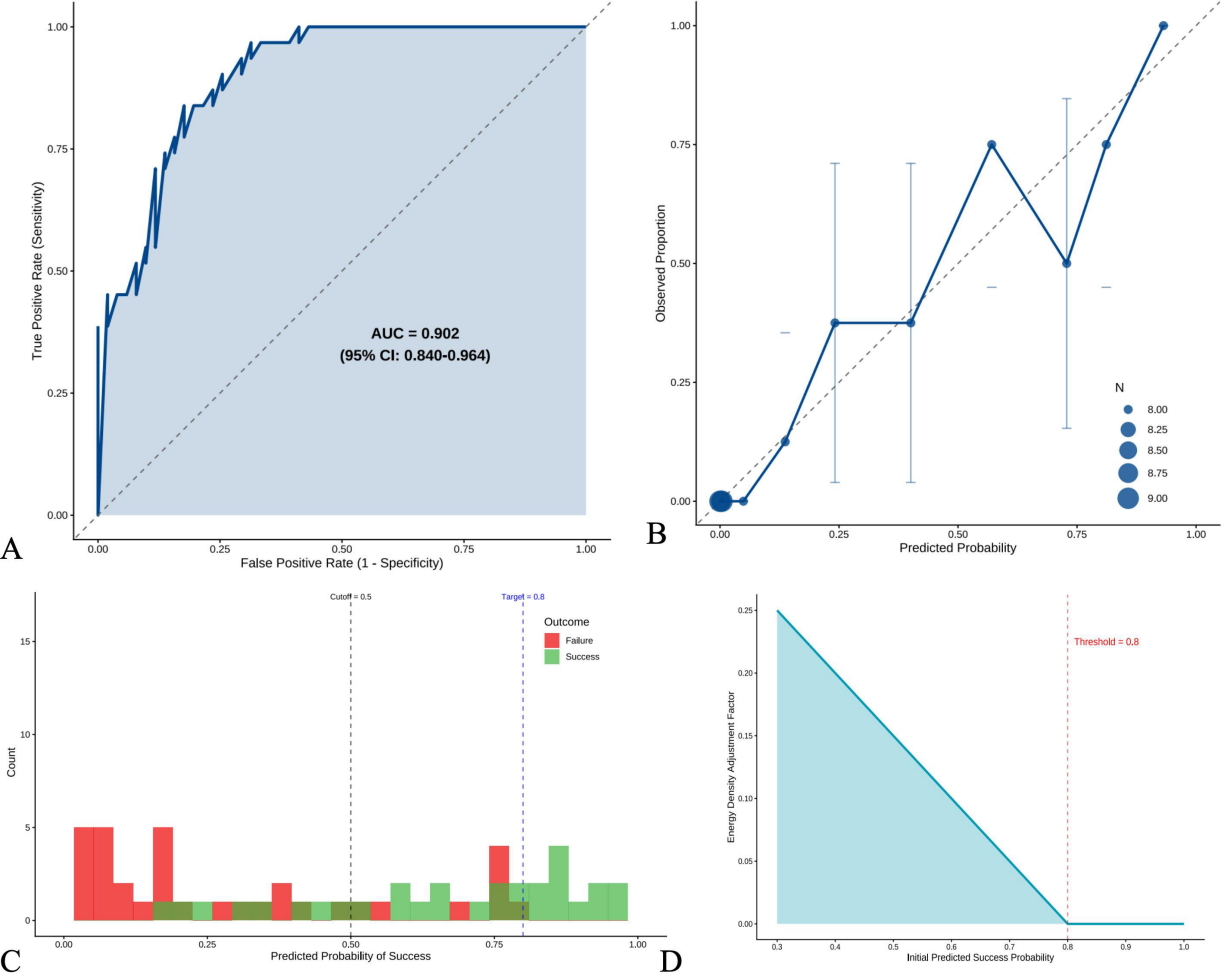
Model performance and validation. **(A)** Receiver operating characteristic (ROC) curve for predicting treatment success (VRR >90% at 12 months). AUC = 0.902 (95% CI: 0.840-0.964). **(B)** Calibration plot comparing predicted versus observed probabilities of treatment success. Diagonal line represents perfect calibration. **(C)** Distribution of predicted probabilities by actual treatment outcome. **(D)** Decision curve analysis showing the optimal probability threshold (0.417) for clinical decision-making.

The treatment success prediction model demonstrated excellent discriminatory ability with an AUC of 0.902 (95% CI: 0.840-0.964) (**Figure 2A**). Using the Youden index, we identified an optimal cutoff probability of 0.417, achieving 83.9% sensitivity and 82.4% specificity, with positive and negative predictive values of 74.3% and 89.4%, respectively (**Supplementary Table 3**). Model calibration was excellent, as confirmed by the Hosmer-Lemeshow test (*P = 0.888*) and visualized in the calibration plot showing close alignment between predicted and observed probabilities (**Figure 2B**). The distribution of predicted probabilities showed clear separation between treatment success and failure groups (**Figure 2C**), validating the model’s discriminatory capacity. Decision curve analysis demonstrated superior net benefit of our personalized approach across threshold probabilities from 0.2 to 0.8, compared to treating all patients with standard energy density or treating none (**Figure 2D**). This confirmed the clinical utility of our modeling approach for prospective patient selection and treatment planning.

### 3.3 Integration into clinical algorithm

Based on validated models, we developed a three-step personalized energy density algorithm to translate our findings into clinical practice (**Table 5**).

**Table 5:** Three-step algorithm for personalized energy density calculation.

Step	Description	Formula/Criteria
Step 1: Base Energy Density	Calculate baseline ED using linear regression model	ED_base = 121.2653 - 6.4368*Vertical_Diameter + 0.0068*Baseline_Volume + 13.7475*TSH + 0.4403*NEUT - 3.2202*Peak_Intensity
Step 2: Success Probability	Estimate probability using logistic regression model	Logit(P) = 1.8341 + 0.6647*Max_Diameter - 0.2570*Trans_Diameter - 0.3267*Long_Diameter - 0.0004*Baseline_Volume - 0.6245*WBC + 0.0489*LYMPH + 0.3198*CRP - 20.3406*CADYes - 18.4603*Enhancement_Typ eNo enhancement +… (2 more terms) P_success = 1/(1 + exp(-Logit(P)))
Step 3: Energy Adjustment	Adjust ED based on predicted success rate	If P(success) < 0.80: ED(final) = ED(base) × [1 + 0.5 × (0.80 - P(success))] If P(success) ≥ 0.80: ED(final) = ED(base)

ED, energy density (J/mm³); VD, vertical diameter; Vol, volume; TSH, thyroid-stimulating hormone; NEUT, neutrophil count; PI, peak intensity; P_success, probability of achieving VRR >90%.

Step 1 - Baseline Energy Calculation: Using the linear regression equation, baseline energy density is calculated incorporating vertical diameter, baseline volume, TSH, neutrophil count, and peak intensity.

Step 2 - Success Probability Estimation: The logistic regression model estimates probability of achieving VRR >90% based on morphological, laboratory, and enhancement parameters.

Step 3 - Adaptive Energy Adjustment: For patients with predicted success probability <80%, energy density is increased proportionally by a factor of [1 + 0.5 × (0.80 - P_success)], ensuring adequate energy delivery for challenging cases while avoiding overtreatment in favorable scenarios.

This algorithm has been implemented as an interactive web-based calculator (https://kyzytcm.shinyapps.io/ultrasound-energy-calculator/) for real-time clinical application (**Supplementary Figure 2**).

## 4 Discussion

This study establishes a comprehensive predictive framework for microwave ablation outcomes in benign thyroid nodules, developing the first multi-parametric energy density optimization algorithm. Our study achieved a mean volume reduction ratio (VRR) of 83.6 ± 12.0% at 12 months, which is comparable to recent multicenter studies reporting mean VRR of 70.8% at median 109-day follow-up (10) and 76% at 12 months following standardized protocols (11). However, when applying a stringent success criterion (VRR >90%), only 37.8% of patients achieved this threshold, highlighting the need for optimized energy delivery protocols. This discrepancy underscores fundamental limitations in current one-size-fits-all approaches. Our finding that baseline volume demonstrated strong negative correlation (*r = -0.27*) with treatment success aligns with recent evidence that smaller nodules achieve superior outcomes, with volume reduction significantly greater in nodules <30mL versus >30mL *(P = 0.0266*) (12). The development of individualized energy delivery protocols represents a critical advance, particularly given evidence that solid nodules require substantially higher energy density than cystic components (13).

The plateau effect observed above 4.0 J/mm³ provides crucial mechanistic insights into thermal ablation limitations. Recent studies demonstrate that excessive energy delivery fails to improve outcomes, with total energy ranging from 1477-7206J showing similar efficacy patterns (14). This phenomenon likely reflects tissue carbonization and heat sink effects that limit further energy deposition effectiveness. A recent RATED study analyzing learning curves found stable treatment efficacy achieved after 20 procedures with median VRR of 70.8%, but notably, neither baseline volume nor energy delivered predicted treatment success beyond initial experience (15). Our data showing energy density >2.19 J/mm³ improved outcomes aligns with reports that monopolar RFA systems require careful energy titration to avoid complications while achieving adequate ablation zones (16). The observation that excessive energy provides diminishing returns challenges current practices and supports precision-based approaches targeting optimal rather than maximal energy deposition.

Five independent predictors emerged from our multivariable analysis, explaining 52% of variance in optimal energy density. The inverse relationship with vertical diameter (*β = -6.44, P < 0.001*) suggests smaller vertical dimensions require concentrated energy for complete ablation, consistent with geometric modeling showing cylindrical ablation fields of approximately 2cm diameter (17). Baseline volume’s positive correlation (*β = 0.007, P = 0.001*) reflects proportional energy requirements, supported by machine learning models identifying initial volume as the strongest predictor (18). TSH level’s emergence as a significant predictor (*β = 13.75, P = 0.044*) represents a novel finding potentially reflecting metabolic activity influencing tissue perfusion and heat dissipation. The neutrophil count association (*β = 0.44, P = 0.009*) suggests baseline inflammatory status affects thermal conductivity, paralleling observations in other thermal ablation contexts (19).

CEUS enhancement patterns demonstrated remarkable predictive value, with hypo-enhancement achieving 41.9% success rate versus 13.7% in failures (*P = 0.004*). These finding parallels recent validation studies showing hypo-enhancement predicts favorable ablation outcomes through reduced perfusion-mediated heat dissipation (20). Iso-enhancement showed markedly reduced success odds (*OR = 0.06, 95% CI: 0.01-0.41*), suggesting enhanced vascularity compromises thermal coagulation through increased heat sink effects. Peak intensity’s negative correlation (*β = -3.22, P = 0.037*) further supports vascular perfusion as a critical determinant of ablation efficacy. These findings align with consensus guidelines recommending CEUS for pre-procedural planning and post-ablation assessment (21).

Inflammatory markers showed unexpected associations with treatment outcomes. Elevated CRP (*OR = 1.38, 95% CI: 1.09-1.73, P = 0.007*) independently predicted success, contrasting with cardiac ablation literature where higher CRP predicts recurrence (22). Reduced WBC count (*OR = 0.54, 95% CI: 0.31-0.93, P = 0.028*) similarly predicted favorable outcomes. These paradoxical findings suggest tissue-specific inflammatory responses in thyroid versus other organs, potentially reflecting underlying nodule characteristics affecting both baseline inflammation and thermal susceptibility. The lymphocyte count trend (*P = 0.111*) warrants further investigation given emerging evidence linking immune responses to ablation outcomes.

Our three-step personalized algorithm addresses critical gaps in current practice. Step 1 calculates baseline energy density incorporating morphological and biochemical parameters. Step 2 estimates success probability using logistic regression with excellent discrimination (*AUC = 0.902*). Step 3 implements adaptive adjustment, increasing energy density proportionally when predicted success falls below 80%. This approach optimizes treatment planning while avoiding overtreatment in favorable cases. Recent artificial intelligence models achieving similar predictive performance validate the feasibility of algorithmic treatment planning (23). The algorithm’s clinical utility extends beyond individual treatment optimization, potentially stratifying patients for alternative therapies when success probability remains low despite energy adjustment.

Several limitations warrant consideration. The retrospective single-center design limits generalizability, particularly given operator experience influences outcomes as demonstrated in learning curve analyses (23). Sample size of 82 patients, while adequate for model development, requires external validation in larger multicenter cohorts. Absence of operator experience quantification represents a significant unmeasured confounder, given evidence that procedural volume affects outcomes. The 12-month follow-up, while standard, may miss late recurrences occurring at 3+ years post-ablation. Additionally, our stringent success definition (>90% VRR) exceeds typical clinical thresholds, potentially underestimating clinical effectiveness.

## 5 Conclusion

In conclusion, this study demonstrates that individualized energy density calculation has the potential to significantly enhance microwave ablation outcomes for benign thyroid nodules. The integration of morphological, biochemical, and vascular parameters into a comprehensive predictive algorithm represents a paradigm shift from standardized to personalized ablation protocols. Prospective multicenter validation studies are warranted to confirm the clinical efficacy of this algorithm and explore additional predictive biomarkers and long-term outcomes beyond 12 months.

## Data Availability

The data that support the findings of this study are available from the corresponding author upon reasonable request. Requests to access the datasets should be directed to LL, linling1980126@163.com.
