# Correction: Development and validation of a multi-parametric energy density optimization algorithm for microwave ablation of benign thyroid nodules: a retrospective cohort study

**DOI:** 10.3389/fendo.2026.1885540

**Published:** 2026-06-03

**Authors:** Mingfeng Mao, Ling Jiang, Xuejing Zhang, Hao Sun, Ling Lin

**Affiliations:** 1 Department of Medical Ultrasonics, The Central Hospital of Wuhan, Tongji Medical College, Huazhong University of Science and Technology, Wuhan, Hubei, China; 2 Department of Otolaryngology, The Central Hospital of Wuhan, Tongji Medical College, Huazhong University of Science and Technology, Wuhan, Hubei, China

**Keywords:** energy density, microwaves ablation techniques, optimization algorithm, precision medicine, thyroid nodule

In the published article, the **Funding** statement was erroneously omitted. The correct **Funding** statement is:

“The author(s) declared that financial support was received for this work and/or its publication. This work was supported by the Wuhan Municipal Natural Science Foundation — Key Special Project for Clinical Research of Municipal Medical Institutions (Grant No. 2026020301040126).”

The original version of this article has been updated.

