# Supplementary material for: Development and validation of a multi-parametric energy density optimization algorithm for microwave ablation of benign thyroid nodules: a retrospective cohort study
Source: Front Endocrinol (Lausanne). 2026 Feb 13;17:1746874. doi: 10.3389/fendo.2026.1746874 (PMC12945780; doi:10.3389/fendo.2026.1746874)
Supplement: Supplementary file 1 [file DataSheet1.docx]

**Supplemental Table 1. Treatment parameters and outcomes (N=82)**

| **Parameters** | **Value** |
| --- | --- |
| **Ablation settings** |  |
| Power (W)ᵃ | 29.5 ± 2.8 |
| Total ablation time (s)ᵃ | 274.7 ± 222.6 |
| Total energy delivered (J)ᵇ | 6300 (2805-12030) |
| Energy density (J/mm³)ᵇ | 2.19 (0.99-24.89) |
| **Treatment outcomes** |  |
| VRR at 3 months (%)ᵃ | 76.1 ± 9.7 |
| VRR at 6 months (%)ᵃ | 77.0 ± 4.4 |
| VRR at 12 months (%)ᵃ | 83.6 ± 12.0 |
| Success rate (VRR >90% at 12 months), n (%) | 31 (37.8) |
| Complications, n (%) | 20 (24.4) |

ᵃData are presented as mean ± standard deviation

**Supplemental Table 2. Comprehensive univariate analysis of baseline characteristics and their association with treatment success**

| **Variable** | **Total (n=82)** | **Insufficient Group (n=51)** | **Success Group (n=31)** | **P-value** |
| --- | --- | --- | --- | --- |
| **Demographics and Anthropometrics** | | | | |
| Age (years) | 49.18 ± 12.29 | 49.86 ± 12.27 | 48.06 ± 12.46 | 0.524 |
| Male sex, n (%) | 10 (12.2) | 7 (13.7) | 3 (9.7) | 0.845 |
| BMI (kg/m²) | 22.30 ± 4.19 | 22.41 ± 4.53 | 22.12 ± 3.64 | 0.769 |
| Weight (kg) | 60.46 ± 9.31 | 60.43 ± 9.61 | 60.52 ± 8.94 | 0.969 |
| **Nodule Characteristics** | | | | |
| Maximum diameter (mm) | 19.02 ± 13.81 | 21.82 ± 14.99 | 14.41 ± 10.24 | **0.017** |
| Transverse diameter (mm) | 15.81 ± 12.22 | 18.49 ± 13.42 | 11.41 ± 8.42 | **0.010** |
| Longitudinal diameter (mm) | 16.62 ± 12.74 | 19.39 ± 14.17 | 12.06 ± 8.34 | **0.011** |
| Vertical diameter (mm) | 13.39 ± 9.78 | 14.93 ± 10.18 | 10.85 ± 8.66 | 0.066 |
| Aspect ratio | 1.00 ± 0.00 | 1.00 ± 0.00 | 1.00 ± 0.00 | -- |
| Baseline volume (mm³) | 8804.91 ± 14253.30 | 11985.10 ± 16685.37 | 3572.99 ± 6248.34 | **0.009** |
| **Thyroid Function Tests** | | | | |
| FT3 (pmol/L) | 4.49 ± 0.62 | 4.55 ± 0.62 | 4.39 ± 0.62 | 0.246 |
| FT4 (pmol/L) | 13.31 ± 1.79 | 13.50 ± 1.72 | 12.98 ± 1.89 | 0.205 |
| TSH (mIU/L) | 1.45 ± 0.73 | 1.41 ± 0.73 | 1.51 ± 0.74 | 0.540 |
| TT3 (ng/dL) | 1.24 ± 0.29 | 1.24 ± 0.28 | 1.24 ± 0.30 | 0.987 |
| TT4 (μg/dL) | 61.58 ± 8.18 | 61.75 ± 8.78 | 61.29 ± 7.20 | 0.807 |
| TGAb (IU/mL) | 49.70 ± 151.44 | 38.93 ± 143.60 | 67.42 ± 164.42 | 0.412 |
| TPOAb (IU/mL) | 66.19 ± 200.50 | 59.14 ± 199.84 | 77.78 ± 204.34 | 0.686 |
| TG (ng/mL) | 19.16 ± 14.85 | 19.30 ± 14.76 | 18.94 ± 15.24 | 0.917 |
| **Laboratory Parameters** | | | | |
| WBC (×10⁹/L) | 6.22 ± 6.20 | 6.79 ± 7.77 | 5.27 ± 1.26 | 0.284 |
| Platelet count (×10⁹/L) | 229.27 ± 40.15 | 233.65 ± 43.60 | 222.06 ± 33.14 | 0.207 |
| Hemoglobin (g/L) | 136.01 ± 12.86 | 136.65 ± 11.50 | 134.97 ± 14.99 | 0.570 |
| Neutrophil count (×10⁹/L) | 21.80 ± 28.11 | 19.11 ± 26.80 | 26.23 ± 30.05 | 0.269 |
| Monocyte count (×10⁹/L) | 1.48 ± 2.38 | 1.27 ± 2.16 | 1.83 ± 2.70 | 0.301 |
| Lymphocyte count (×10⁹/L) | 9.99 ± 15.25 | 7.89 ± 13.12 | 13.43 ± 17.92 | 0.111 |
| CRP (mg/L) | 4.05 ± 4.32 | 3.51 ± 3.35 | 4.93 ± 5.51 | 0.151 |
| **CEUS Parameters** | | | | |
| Peak intensity | 25.41 ± 3.63 | 25.06 ± 3.78 | 25.98 ± 3.34 | 0.270 |
| Time to peak (s) | 19.76 ± 4.11 | 19.32 ± 4.06 | 20.48 ± 4.15 | 0.218 |
| Slope | 1.88 ± 0.93 | 1.98 ± 1.05 | 1.72 ± 0.70 | 0.220 |
| AUC of contrast curve | 1508.79 ± 210.44 | 1510.20 ± 209.99 | 1506.48 ± 214.64 | 0.939 |
| **Comorbidities, n (%)** |  |  |  |  |
| Hypertension | 14 (17.1) | 8 (15.7) | 6 (19.4) | 0.900 |
| Diabetes mellitus | 0 (0.0) | 0 (0.0) | 0 (0.0) | -- |
| Coronary artery disease | 2 (2.4) | 2 (3.9) | 0 (0.0) | 0.705ᵃ |
| **Imaging Features, n (%)** | | | | |
| TIRADS 4 | 18 (22.0) | 9 (17.6) | 9 (29.0) | 0.351 |
| Unclear margin | 2 (2.4) | 1 (2.0) | 1 (3.2) | 1.000ᵃ |
| Regular shape | 82 (100.0) | 51 (100.0) | 31 (100.0) | -- |
| Solitary nodule | 31 (37.8) | 19 (37.3) | 12 (38.7) | 1.000 |
| **Nodule Composition, n (%)** | | | | 0.504 |
| Mixed cystic-solid | 15 (18.3) | 10 (19.6) | 5 (16.1) |  |
| Cystic | 9 (11.0) | 7 (13.7) | 2 (6.5) |  |
| Solid | 58 (70.7) | 34 (66.7) | 24 (77.4) |  |
| **Location, n (%)** |  |  |  | 0.567 |
| Bilateral | 9 (11.0) | 6 (11.8) | 3 (9.7) |  |
| Right lobe | 33 (40.2) | 23 (45.1) | 10 (32.3) |  |
| Right lobe lower segment | 1 (1.2) | 1 (2.0) | 0 (0.0) |  |
| Right lobe near isthmus | 1 (1.2) | 1 (2.0) | 0 (0.0) |  |
| Isthmus | 1 (1.2) | 0 (0.0) | 1 (3.2) |  |
| Left side | 6 (7.3) | 3 (5.9) | 3 (9.7) |  |
| Left lobe | 31 (37.8) | 17 (33.3) | 14 (45.2) |  |
| Calcification present | 24 (29.3) | 18 (35.3) | 6 (19.4) | 0.198 |
| Dangerous triangle location | 35 (42.7) | 21 (41.2) | 14 (45.2) | 0.902 |
| **Enhancement Pattern, n (%)** | | | | **0.004** |
| Hypoenhancement | 20 (24.4) | 7 (13.7) | 13 (41.9) |  |
| No enhancement | 7 (8.5) | 7 (13.7) | 0 (0.0) |  |
| Isoenhancement | 26 (31.7) | 20 (39.2) | 6 (19.4) |  |
| Hyperenhancement | 29 (35.4) | 17 (33.3) | 12 (38.7) |  |
| Enhancement defect present | 58 (70.7) | 37 (72.5) | 21 (67.7) | 0.831 |
| Enhancement ring present | 16 (19.5) | 11 (21.6) | 5 (16.1) | 0.752 |

Data are presented as mean ± standard deviation or n (%) unless otherwise specified
Bold values indicate statistical significance (P < 0.05)
ᵃFisher's exact test
Success defined as VRR >90% at 12 months
Abbreviations: BMI, body mass index; CEUS, contrast-enhanced ultrasound; CRP, C-reactive protein; FT3, free triiodothyronine; FT4, free thyroxine; TG, thyroglobulin; TGAb, thyroglobulin antibody; TIRADS, Thyroid Imaging Reporting and Data System; TPOAb, thyroid peroxidase antibody; TSH, thyroid-stimulating hormone; TT3, total triiodothyronine; TT4, total thyroxine; VRR, volume reduction ratio; WBC, white blood cell count

**Supplemental Table 3. Performance metrics of the prediction model**

| **Metric** | **Value** |
| --- | --- |
| Area under the curve (AUC) | 0.902 |
| 95% Confidence interval | 0.840-0.964 |
| Optimal cutoff probability | 0.417 |
| Sensitivity | 83.9% |
| Specificity | 82.4% |
| Positive predictive value | 74.3% |
| Negative predictive value | 89.4% |
| Hosmer-Lemeshow test P-value | 0.888 |

**
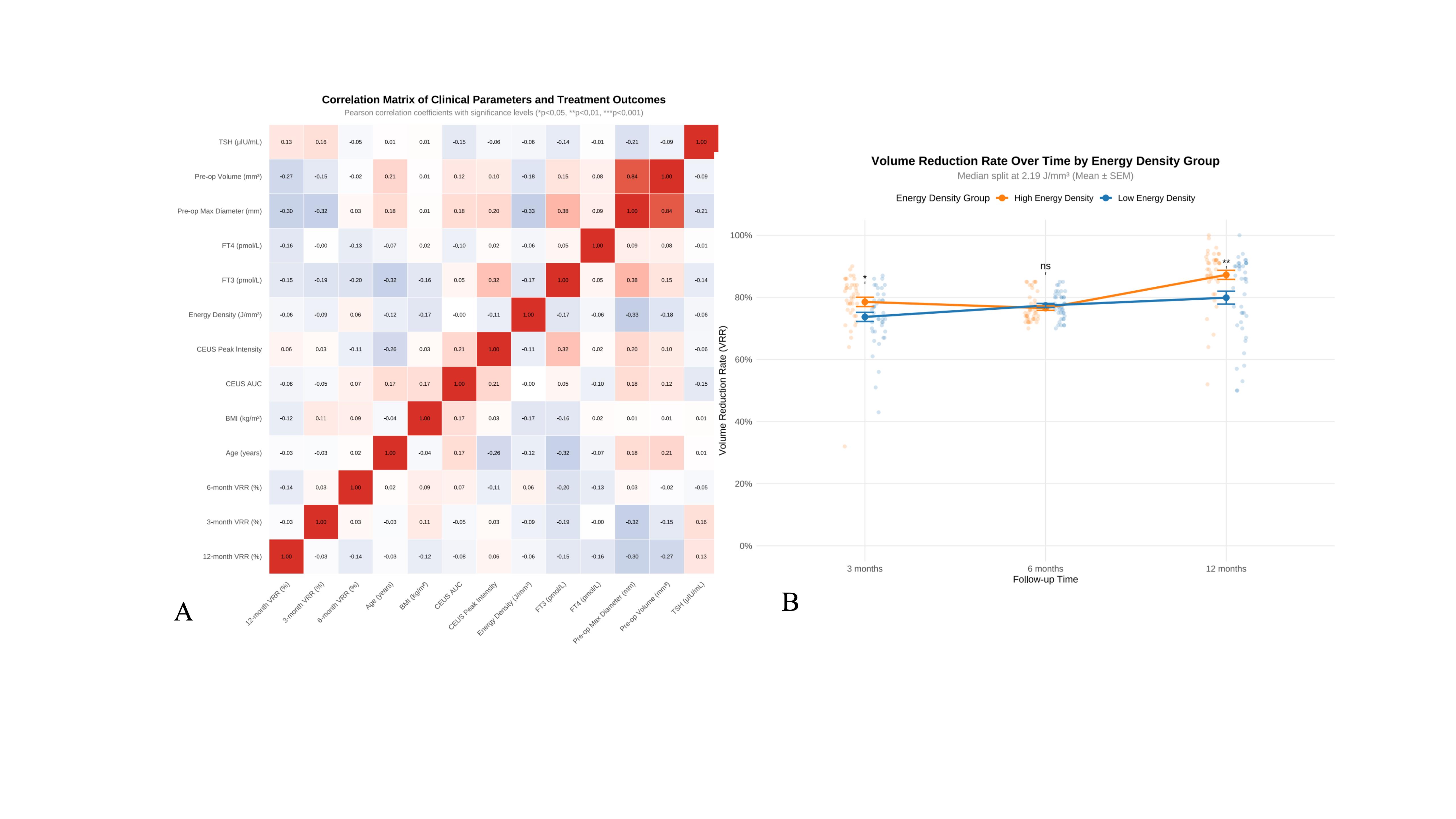
**

**Supplemental Figure 1. Clinical predictors and treatment outcomes analysis**

(A) Heatmap showing correlation between clinical characteristics and treatment success. Red indicates positive correlation, blue indicates negative correlation. (B) Comparison of volume reduction ratio trends between high energy density (>2.19 J/mm³) and low energy density (≤2.19 J/mm³) groups over 12-month follow-up period. Error bars represent standard error of the mean.


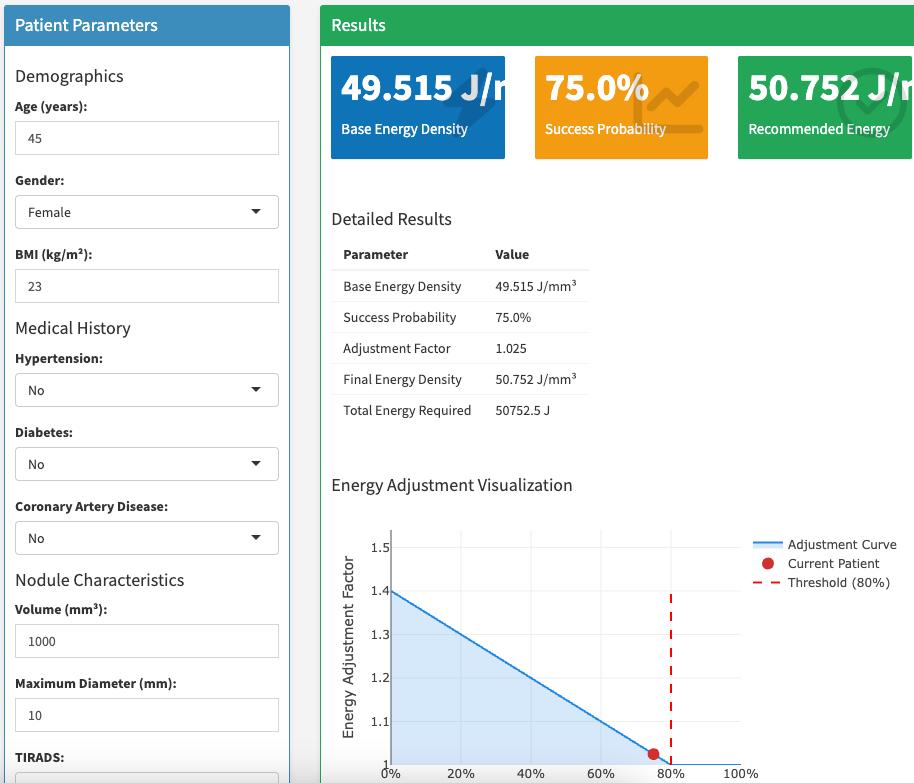


**Supplemental Figure 2. Interactive web-based calculator**
